# Outcome Comparison of Acute Respiratory Distress Syndrome (ARDS) in Patients with Trauma-Associated and Non-Trauma-Associated ARDS: A Retrospective 11-Year Period Analysis

**DOI:** 10.3390/jcm11195734

**Published:** 2022-09-28

**Authors:** Lilian Jo Engelhardt, Claudio Olbricht, Marcel Niemann, Jan Adriaan Graw, Oliver Hunsicker, Björn Weiss, Victoria Bünger, Steffen Weber-Carstens, Sebastian Daniel Boie, Sophie K. Piper, Felix Balzer, Mario Menk

**Affiliations:** 1 Department of Anesthesiology and Operative Intensive Care Medicine (CCM/CVK), Charité—Universitätsmedizin Berlin, Corporate Member of Freie Universität Berlin and Humboldt-Universität zu Berlin, Augustenburger Platz 1, 13353 Berlin, Germany or; 2 Institute of Medical Informatics, Charité—Universitätsmedizin Berlin, Corporate Member of Freie Universität Berlin and Humboldt-Universität zu Berlin, Charitéplatz 1, 10117 Berlin, Germany; 3 Klinik für Anästhesie und Intensivmedizin, Evangelische Elisabeth Klinik Johannesstift Diakonie, Lützowstraße 24–26, 10785 Berlin, Germany; 4 Center for Musculoskeletal Surgery, Charité—Universitätsmedizin Berlin, Corporate Member of Freie Universität Berlin and Humboldt-Universität zu Berlin, Augustenburger Platz 1, 13353 Berlin, Germany; 5 Julius Wolff Institute for Biomechanics and Musculoskeletal Regeneration, Berlin Institute of Health at Charité—Universitätsmedizin Berlin, Augustenburger Platz 1, 13353 Berlin, Germany; 6 Institute of Biometry and Clinical Epidemiology, Charité—Universitätsmedizin Berlin, Corporate Member of Freie Universität Berlin and Humboldt-Universität zu Berlin, Charitéplatz 1, 10117 Berlin, Germany

**Keywords:** acute respiratory distress syndrome, trauma, multiply injured patients, extracorporeal membrane oxygenation, ICU, critical illness, outcome, mortality

## Abstract

(1) Background: Acute respiratory distress syndrome (ARDS) is a rare complication in multiply injured patients. Due to the rarity of ARDS development after trauma, little is known about outcomes of patients with trauma-associated ARDS compared to patients with non-trauma-associated ARDS. (2) Methods: This retrospective analysis included *n* = 1038 ARDS patients admitted to the ARDS center of Charité—Universitätsmedizin Berlin between 2007 and 2018. Patients with trauma-associated ARDS (*n* = 62) were compared to patients with non-trauma-associated ARDS (*n* = 976). In a secondary analysis, patients from the group with non-trauma-associated ARDS were 1:1 nearest neighbor matched to patients with trauma-associated ARDS. The primary outcomes were 28-day in-hospital mortality, 60-day in-hospital mortality, and overall in-hospital mortality. (3) Results: Overall in-hospital mortality in trauma-associated ARDS was 29.0% compared to 40.5% in all patients with non-trauma-associated ARDS (*p* = 0.074). The in-hospital mortality rate in matched patients with non-trauma-associated ARDS (33.9%) was comparable to the trauma-associated ARDS cohort (*p* = 0.701). Kaplan–Meier curves indicated time-sensitive variations in 28-day and 60-day in-hospital survival. (4) Conclusion: Mortality was not different in patients with trauma-associated ARDS compared to patients with non-trauma-associated ARDS. Survival rate in the Kaplan–Meier curves stabilized after the critical initial phase and throughout the further 60-day period in patients with trauma-associated ARDS compared to patients with non-trauma-associated ARDS. Since this divergence was less pronounced in the matched cohort, it may be related to the younger age, fewer comorbidities, and lower ARDS severity in patients with trauma-associated ARDS. Patients with trauma-associated ARDS remain a very different cohort compared to patients with non-trauma-associated ARDS. Therefore, the outcome comparison is limited, even after matching.

## 1. Introduction

Acute respiratory distress syndrome (ARDS) has recently gained public attention in the context of the COVID-19 pandemic [1]. Apart from infectious origin, ARDS can be induced by multiple causes, including trauma [2]. In multiply injured patients, ARDS is a rare complication. A decrease in incidence of ARDS (3 to 1.1%) has recently been reported in an investigation of trauma patients from the national Trauma Quality Improvement Program dataset [3]. Risk factors for ARDS after multiple trauma include traumatic brain and chest injury, severity and duration of shock, number of transfused blood products, and infused crystalloids [2,4,5]. Clinical management of trauma-associated ARDS poses a particular challenge to intensive care unit (ICU) staff. For example, prone positioning may be contraindicated in patients with brain injury and increased intracerebral pressure [6,7]. In addition, therapeutic anticoagulation for therapy with extracorporeal membrane oxygenation (ECMO) is associated with the risk of hemorrhage [6], which may aggravate clinical course and complicate injury patterns.

ARDS in general is associated with a high mortality and it is a relevant topic in the ICU [8]. Survivors of the complex lung pathology can suffer from major long-term consequences, such as post-intensive care syndrome [9]. Since ARDS has a wide range of causes, its cohort characteristics, course of disease, and outcome can be very different. Due to the rarity of ARDS development after trauma, little is known about clinical course and outcomes, particularly in comparison to non-trauma-associated ARDS.

Several previous studies reported variable mortality rates in traumatic ARDS [10]. In a recent investigation of the Trauma Quality Improvement Program database, a tendency to increased mortality of traumatic ARDS has been reported in the period between 2010 and 2014 [3].

A better understanding of specific sub-cohorts, such as patients with trauma-associated ARDS is required. The goal of this retrospective analysis was to investigate the outcome of individuals with trauma-associated ARDS compared to a large cohort of patients with non-trauma-associated ARDS treated in a tertiary ARDS referral center, specifically focusing on mortality and utilizing nearest neighbor matching to minimize potential confounders and provide comparability.

## 2. Materials and Methods

### 2.1. Inclusion Criteria, Setting and Data Source

The study includes data from adult patients (≥18 years) with ARDS admitted to the national ARDS referral center of the Department of Anesthesiology and Operative Intensive Care Medicine, Charité—Universitätsmedizin Berlin between 2007 and 2018. ARDS was defined according to the 2012 Berlin definition, including acute onset of hypoxemia within one week, paO_2_/FiO_2_ ratio of <300 mmHg and PEEP >5 cmH_2_O, respiratory failure not fully explained by cardiac cause or fluid overload, and bilateral opacities [11]. Clinical routine data were extracted from the local patient data management system (COPRA, Sasbachwalden, Germany and SAP, Walldorf, Germany), according to previously published data [12]. The analysis was approved by the Ethics Committee, Ethikkommission Charité.

### 2.2. Outcome Measures and Variables of Interest

The primary outcome parameters were 28-day in-hospital mortality, 60-day in-hospital mortality, and overall in-hospital mortality. Cumulative survival until day 28 and day 60 in the hospital was illustrated in Kaplan–Meier plots.

Secondary outcomes were details of mechanical ventilation, pulmonary gas exchange, and supportive therapies, as well as number of transfused units of packed red blood cells, fresh frozen plasma (FFP), and platelets. Further variables of interest included injury severity and multiorgan dysfunction scores.

### 2.3. Group Categorization and Matching

**Figure 1:**
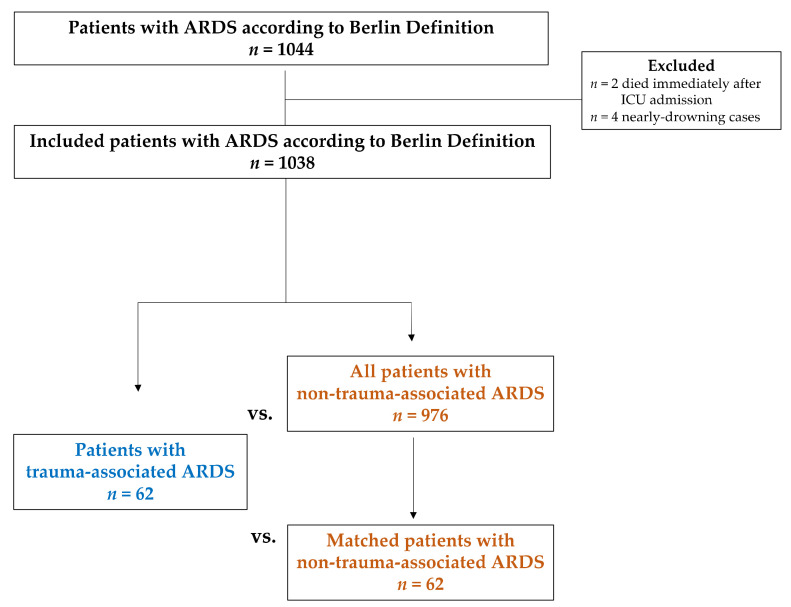
Flow chart of the study cohort and groups.

Figure 1 illustrates the study cohort and groups in a flow chart. Patients who developed ARDS after trauma were categorized as trauma-associated ARDS. Patients admitted because of trauma who developed ARDS several days afterwards were also classified as trauma-associated ARDS. ARDS induced by all other pathomechanisms was classified as non-trauma-associated ARDS. Near-drownings were excluded.

Firstly, the outcome parameters of patients with trauma-associated ARDS were compared to all patients with non-trauma-associated ARDS. For a secondary analysis, patients from the non-trauma-associated ARDS group were 1:1 nearest neighbor matched to trauma-associated ARDS patients to account for selection bias. The matching was performed manually, based on the parameters at ICU admission. The variables age, sex, sequential organ failure assessment (SOFA) score, and oxygenation index were taken into consideration. Tolerance regions included five years for age, five points for SOFA score and eight points for oxygenation index. If the oxygenation index was not available, Berlin ARDS definition [11] was used as alternative matching variable. Patients with ECMO were classified as patients with severe ARDS.

Secondly, the primary outcome of patients with extracorporeal lung support was investigated exclusively in a sub-analysis. Extracorporeal lung support (ECLS) summarized patients with ECMO and extracorporeal lung assist (ECLA).

### 2.4. Statistical Analysis

For descriptive statistical analysis, SPSS (SPSS^®^ Statistics, Version 27, IBM Corp. Released 2020, Armonk, NY) was used. Kaplan–Meier plots were generated by Python (Version 3.8.5) and Panda (Version 1.3.5). Results were expressed as median with limits of the interquartile range (25th; 75th percentile) or as absolute numbers with percentages. For the comparison between patients with trauma-associated ARDS and all patients with non-trauma-associated ARDS (independent groups), the Mann–Whitney U test and Pearson’s Chi^2^ were applied as non-parametric tests. For the group comparison of patients with trauma-associated ARDS and matched patients with non-trauma-associated ARDS (dependent groups), the Wilcoxon signed-rank test was applied for numerical variables and McNemar’s Test for categorical variables. The 28-day and 60-day cumulative survival was illustrated by Kaplan–Meier curves with 95% confidence intervals. The Tarone–Ware test was applied to compare survival rates between groups. All statistical tests constitute exploratory analysis without adjustment for multiple comparisons. A *p*-value ≤ 0.05 was considered statistically significant.

## 3. Results

Overall, *n* = 1044 ARDS patients were treated in the ARDS center of the Department of Anesthesiology and Operative Intensive Care Medicine of Charité—Universitätsmedizin Berlin in the 11-year period from 2007 to 2018. The flow chart with included patients and groups is illustrated in Figure 1. Two patients who died immediately after ICU admission and four patients with a near-drowning incident were excluded. A total of *n* = 1038 ARDS patients were included in this analysis. More than 90% of patients in each group already presented with ARDS at ICU admission, since the study setting is an ARDS referral center.

*n* = 62 patients developed ARDS after an initial traumatic incident, classified as trauma-associated ARDS. One patient with trauma-associated ARDS developed ARDS after ICU admission due to aspiration. All other patients with trauma-associated ARDS presented with ARDS at ICU admission.

The primary cause for trauma was traffic accident (59.7%), followed by fall from height of more than 3 m (22.6%). Further reasons were fall from less than 3 m (8.1%), bodily harm (4.8%), and others (3.2%). The etiology of non-trauma-associated ARDS was most frequently related to viral or bacterial pneumonia (67.8%), followed by aspiration (14.1%), others (10%), non-pulmonary sepsis (6.1%), and pancreatitis (1.8%).

### 3.1. Baseline Characteristics

Baseline characteristics are presented in Table 1. Patients with trauma-associated ARDS were younger (*p* = 0.003), were more likely male (*p* = 0.004), and suffered less frequently from chronic diseases (*p* < 0.001) when compared to all patients with non-trauma-associated ARDS (Table 1, Group A vs. B1). When the baseline characteristics of patients with trauma-associated ARDS were compared to the matched sub-group of patients with non-trauma-associated ARDS, chronic diseases represented by Charlson Comorbidity Index remained significantly fewer in patients with trauma-associated ARDS (*p* < 0.001) (Table 1, Group A vs. B2).

**Figure 2:**
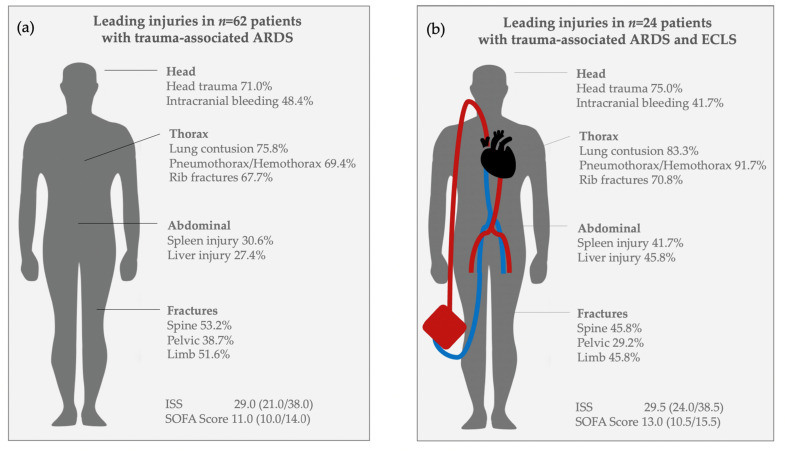
Injury patterns. (**a**) Leading injuries in all patients with trauma-associated ARDS. (**b**) Leading injuries in patients with trauma-associated ARDS and ECLS treatment.

Median Injury Severity Score (ISS) was 29.0 (21.0/38.0). Details of injury patterns of all patients with trauma-associated ARDS are shown in Figure 2a, and for the sub-group of patients with trauma-associated ARDS and ECLS treatment in Figure 2b. Lung contusion and head trauma were the leading injuries. Aspiration was suspected in *n* = 14 (22.6%) patients with trauma-associated ARDS.

### 3.2. Primary Outcome

Details on 28-day in-hospital mortality, 60-day in-hospital mortality, and overall in-hospital mortality are shown in Table 2.

#### 3.2.1. Mortality (28-Day In-Hospital, 60-Day In-Hospital, and Overall In-Hospital)

Twenty-eight-day in-hospital mortality, 60-day in-hospital mortality, and overall in-hospital mortality showed no significant differences in the individuals with trauma-associated ARDS compared to all patients with non-trauma-associated ARDS (Table 2a, Group A vs. B1). In the matched cohort, 28-day, 60-day, and in-hospital mortality were also comparable between patients with trauma-associated ARDS and matched patients with non-trauma-associated ARDS (Table 2a, Group A vs. B2).

#### 3.2.2. Mortality (28-Day In-Hospital Mortality, 60-Day In-Hospital Mortality, and Overall In-Hospital) in Patients with Extracorporeal Lung Support (ECLS) Treatment

In the sub-analysis exclusively on patients with ECLS treatment, the 28-day in hospital-mortality, 60-day in-hospital mortality, and overall in-hospital mortality rates showed no significant differences in patients with trauma-associated ARDS and ECLS treatment compared to all patients with non-trauma-associated ARDS and ECLS treatment (Table 2b, Group A vs. B1). When patients with trauma-associated ARDS and ECLS treatment were compared to the matched cohort of patients with non-trauma-associated ARDS and ECLS treatment, 28-day in-hospital mortality, 60-day in-hospital mortality and overall in-hospital mortality rates did not differ between both groups (Table 2b, Group A vs. B2).

#### 3.2.3. Kaplan–Meier Survival Curves (28-Day in-Hospital and 60-Day in-Hospital)

**Figure 3:**
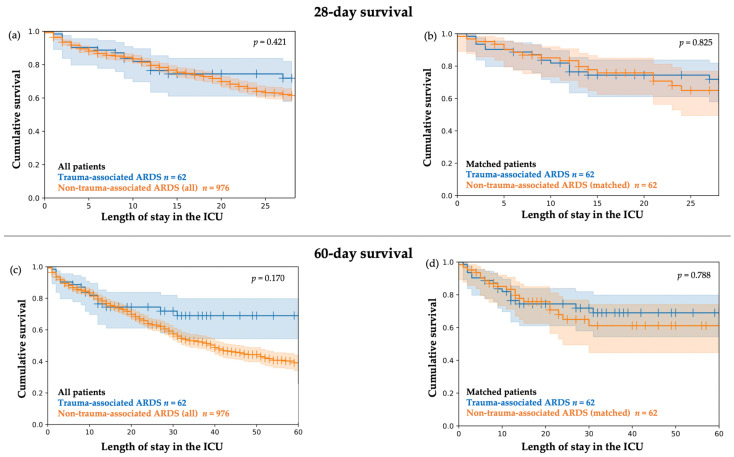
Kaplan–Meier plots. Cumulative 28-day in-hospital survival and 60-day in-hospital survival with 95% CI. (**a**) Cumulative 28-day survival in *n* = 62 patients with trauma-associated ARDS compared to *n* = 976 patients with non-trauma-associated ARDS. Tarone–Ware Test *p* = 0.421 (**b**) Cumulative 28-day survival in *n* = 62 patients with trauma-associated ARDS compared to *n* = 62 matched patients with non-trauma-associated ARDS. Tarone–Ware Test *p* = 0.825. (**c**) Cumulative 60-day in-hospital survival in *n* = 62 patients with trauma-associated ARDS compared to *n* = 976 patients with non-trauma-associated ARDS. Tarone–Ware Test *p* = 0.170 (**d**) Cumulative 60-day in-hospital survival in *n* = 62 patients with trauma-associated ARDS compared to *n* = 62 matched patients with non-trauma-associated ARDS. Tarone–Ware Test *p* = 0.788.

The 28-day in-hospital survival and 60-day in-hospital survival (illustrated by Kaplan–Meier plots) in patients with trauma-associated ARDS compared to all patients with non-trauma-associated showed initial similar courses and started to diverge between day 15 and day 20, although the differences were not significant (Figure 3a,c). In addition, 28-day in-hospital survival and 60-day in-hospital survival rates were similar between patients with trauma-associated ARDS and the matched patients with non-trauma-associated ARDS (Figure 3b,d).

#### 3.2.4. Kaplan–Meier Survival Curves (28-Day In-Hospital Survival and 60-Day In-Hospital Survival) in Patients with Extracorporeal Lung Support (ECLS)

**Figure 4:**
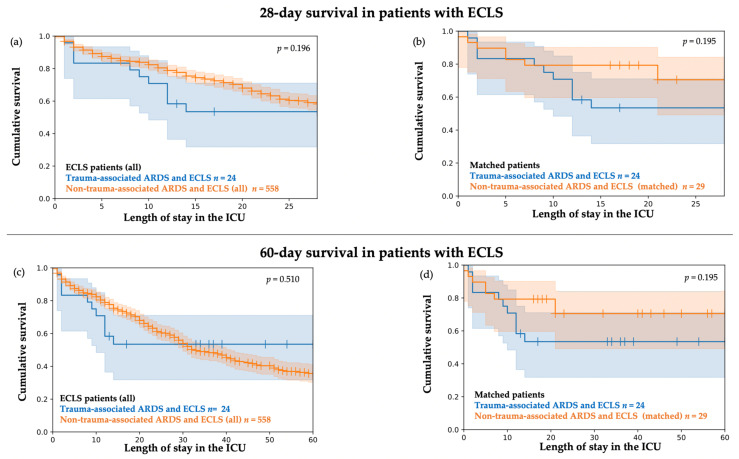
Kaplan–Meier plots. Cumulative 28-day and 60-day survival with 95% CI in the sub-cohort of patients with extracorporeal lung support (ECLS). (**a**) Cumulative 28-day survival in *n* = 24 patients with trauma-associated ARDS and ECLS treatment compared to *n* = 558 patients with non-trauma-associated ARDS and ECLS treatment. Tarone–Ware Test *p* = 0.196. (**b**) Cumulative 28-day survival in *n* = 24 patients with trauma-associated ARDS and ECLS treatment compared to *n* = 29 matched patients with non-trauma-associated ARDS and ECLS treatment. Tarone–Ware Test *p* = 0.195. (**c**) Cumulative 60-day survival in *n* = 24 patients with trauma-associated ARDS and ECLS treatment compared to *n* = 558 patients with non-trauma-associated ARDS and ECLS treatment. Tarone–Ware Test *p* = 0.510 (**d**) Cumulative 60-day survival in *n* = 24 patients with trauma-associated ARDS and ECLS treatment compared to *n* = 29 matched patients with non-trauma-associated ARDS and ECLS treatment. Tarone–Ware Test *p* = 0.195.

The probability of cumulative 28-day in-hospital survival and 60-day in-hospital survival by Kaplan–Meier plots for the sub-cohort of patients with ECLS treatment is illustrated in Figure 4.

In the sub-analysis exclusively on patients with ECLS treatment, the Kaplan–Meier curves started to diverge after the first month in patients with trauma-associated ARDS and ECLS treatment compared to all patients with non-trauma-associated ARDS and ECLS treatment (Figure 4a,c). The probability of cumulative 28-day in-hospital survival and 60-day in-hospital survival was not significantly different.

In addition, the probability of cumulative 28-day in-hospital survival and 60-day in-hospital survival did not differ significantly between patients with trauma-associated ARDS and ECLS treatment and the matched sub-group of patients with non-trauma-associated ARDS and ECLS treatment (Figure 4b,d).

### 3.3. Secondary Outcomes

#### 3.3.1. Mechanical Ventilation, Pulmonary Gas Exchange, and Supportive Therapies

Details of mechanical ventilation, pulmonary gas exchange, and supportive therapies are shown in Table 3. All patients received mechanical ventilation.

Patients with trauma-associated ARDS suffered less frequently from severe ARDS (*p* < 0.001), were ventilated less invasively, and presented with better pulmonary gas exchange parameters compared to all patients with non-trauma-associated ARDS. In accordance, patients with trauma-associated ARDS were less frequently treated with ECLS (*p* = 0.005), NO inhalation (*p* = 0.049) and prone positioning (*p* = 0.036). (Table 3, Group A vs. B1). No patient with trauma-associated ARDS received primary veno-arterial ECMO.

When patients with trauma-associated ARDS were compared to the matched sub-group of patients with non-trauma-associated ARDS, the P_a_CO_2_ in the first 6 h after ICU admission remained significantly lower in patients with trauma-associated ARDS (*p* = 0.046) (Table 3, Group A vs. B2).

#### 3.3.2. Transfusions

Patients with trauma-associated ARDS received in median 9 more units of fresh frozen plasma (*p* = 0.015), 2.5 (*p* = 0.099) more units of packed red blood cells, and 2.0 (*p* = 0.346) more units of platelet concentrate during ICU stay compared to matched patients with non-trauma-associated ARDS (Table 4, Group A vs B2).

## 4. Discussion

Patients with trauma-associated ARDS presented with different baseline characteristics, such as younger age, less comorbidities, and a lower severity of ARDS compared to all patients with non-trauma-associated ARDS. No significant mortality differences were found in patients with trauma-associated ARDS compared to patients with non-trauma-associated ARDS. This finding was also observed in the sub-groups of patients with ECLS treatment.

Although not significant, time-sensitive differences in survival were indicated by the Kaplan–Meier curves, particularly in the non-matched comparison. Survival was highly similar in patients with trauma-associated ARDS and all patients with non-trauma-associated ARDS in the initial period of ICU stay. The curves started to diverge after the first 15 to 20 days, with a stabilizing survival rate throughout the further 60-day period in patients with trauma-associated ARDS compared to patients with non-trauma-associated ARDS. Since the divergence of the Kaplan–Meier curves was less pronounced in the matched cohort, this was most likely related to the younger age, fewer comorbidities, and lower ARDS severity in patients with trauma-associated ARDS when compared to all patients with non-trauma-associated ARDS. In particular, the initial phase seemed to be critical in patients with trauma-associated ARDS, as indicated by the Kaplan–Meier curves. Early trauma-induced complications and therapy limitations, e.g. due to irreversible intracranial damage and multiorgan failure may have contributed. In patients with trauma-associated ARDS and ECLS treatment, the majority of the deceased patients died during the initial period until day 15. As stated above, therapy limitations due to early infaust prognosis may have played a role. In addition, early bleeding complications might be relevant, especially in anticoagulated patients with trauma-associated ARDS and ECLS treatment.

A recent analysis of the Trauma Quality Improvement Program database from the American College of Surgeons Committee on Trauma included more than 12,000 ARDS patients after trauma. In this nationwide data sample, Kasotakis et al. reported a tendency towards increased in-hospital mortality of patients with ARDS after trauma in the period between 2010 and 2014, reaching a peak of 21% in 2014 [3]. This in-hospital mortality rate was lower compared to the mortality rate of 29.0% described for patients with trauma-associated ARDS in the present analysis. However, the ISS of the patients included in the analysis of the Trauma Quality Improvement Program database was lower compared to the present investigation. Data on severity of ARDS and multiorgan failure were not reported by the authors, and therefore an appropriate comparison between the study cohorts is not possible [3].

Particularly in patients with trauma-associated ARDS and ECLS treatment, a high in-hospital mortality rate of 50.0% was observed in the present analysis. Several retrospective studies previously investigated mortality in trauma patients with ARDS and ECLS treatment, with diverging results [10,13,14,15]. The largest retrospective analysis from the Multicenter Extracorporeal Life Support Organization (ELSO) Registry identified 279 trauma patients treated with ECLS in the period between 1989 and 2016, of which 89% received therapy with veno-venous ECMO for acute respiratory failure [16]. In these patients, survival to hospital discharge was 63% and therefore higher compared to the present analysis [16]. Another analysis of the ELSO Registry included only patients treated with ECMO after blunt thoracic trauma [17]. Here, favorable survival rates to hospital discharge of 74.1% were identified [17]. The investigators concluded that trauma patients are potentially an ideal population for ECMO therapy due to their young age (<30 years), generally few comorbidities, and reversible injuries, if bleeding complications are controllable [17]. However, injury patterns with intracranial hemorrhage were observed in only 16.5% of the patients with ECMO after blunt thoracic trauma in the ELSO Registry patients [17]. This was considerably lower compared to rates of intracranial hemorrhage in patients with trauma-associated ARDS and ECLS treatment in the present analysis (41.7%). Unfortunately, no data on the severity of organ failure were published, which strongly limits further comparison. Moreover, better outcomes in patients with ARDS and treatment with ECLS after trauma were reported from two other German centers [13,14]. Ried et al. and Ull et al. reported in-hospital mortality rates of 21.2% and 34.7%, respectively, although an injury severity score of 58.9 was reported by Ried et al. [13,14]. Severe multiorgan dysfunction at ICU admission indicated by a SOFA score of 13.0 may have contributed to the high mortality rate in patients with trauma-associated ARDS and ECLS treatment in the present analysis. In comparison, Ried et al. observed a SOFA score of 10.5, and Ull et al. a SOFA score of 12.0 in patients with ARDS after trauma and ECLS treatment [13,14]. Moreover, the rate of head trauma (75.0% vs. 58%) and intracranial bleeding at admission (41.7% vs. 26.9%) were higher in patients with trauma-associated ARDS and ECLS treatment in the present analysis, compared to the investigation of Ried et al. [13]. Newly developed intracranial hemorrhage during therapy with veno-venous ECMO has been identified as a determinant for 60-day mortality [18]. Slightly lower survival rates of 44% were reported by Ahmad et al. in patients with veno-venous ECMO therapy after traumatic injury [19]. In polytraumatic patients who received veno-arterial ECMO as rescue therapy, survival rates were lower [20]. Bonacchi et al. reported successful initiation of veno-arterial ECMO in cardiopulmonary failure with refractory shock in 14 patients, with five patients surviving until ICU discharge and seven patients bridging to brain death assessment [20].

A potential contributing mechanism to trauma-associated ARDS may be aspiration, which was suspected in at least 22.6% of the patients in the present analysis. This is in agreement with a prospective investigation of the contamination of vocal cords at the time point of endotracheal intubation, which was observed in 18 (34%) of 53 patients with severe trauma [21]. There might be additional unwitnessed aspirations in the present analysis, particularly in the high number of patients with head injury. Reduced levels of consciousness are obviously a known risk factor for aspiration, though the actual incidence of aspiration-induced lung injury is difficult to estimate [22]. So far it remains unclear to what extent pulmonary aspiration contributed to ARDS development in the patients with trauma-associated ARDS.

In the present analysis, a significantly higher number of units of FFP was transfused in patients with trauma-associated ARDS compared to matched patients with non-trauma-associated ARDS. Transfusion of FFP in the first 24 h after injury was independently associated with the development of ARDS in patients with blunt injury and hemorrhagic shock in a multicenter prospective cohort study published in 2009 [23]. Particularly after transfusion of FFP, the under-recognized potential differential diagnosis of transfusion-associated lung injury (TRALI) needs to be taken into consideration [24]. However, since the exclusion of plasma products from female donors in 2009, the incidence of transfusion-associated lung injury has further decreased [25]. It is unclear whether transfusion of FFP might have contributed to ARDS development in patients with trauma-associated ARDS in this retrospective study. However, considering the observation period from 2007 to 2018 for this analysis, some ARDS patients were treated before 2009 and therefore may have been at increased risk for TRALI [25].

Lower severity of ARDS was observed in patients with trauma-associated ARDS when compared to all patients with non-trauma-associated ARDS in the present analysis. This finding may be related to the observed better baseline characteristics, such as younger age and fewer chronic diseases. Accordingly, mechanical ventilation and supportive therapies were less invasive in patients with trauma-associated ARDS. Although traumatic incidents predominantly affect younger persons, increasing numbers of elderly multiply injured patients can be expected in the future due to the aging population [26]. An epidemiologic study on mortality after trauma in the US population identified trauma as the leading cause of death in persons 46 years and younger in 2010 [26]. Interestingly, the authors already observed a shift to higher ages in the period between 2000 and 2010 [26]. This development may potentially lead to an even more challenging treatment in trauma-associated ARDS in the future.

## 5. Limitations

This is a single-center retrospective and exploratory data analysis. The potential risk of bias in the comparison of patients with trauma-associated ARDS and patients with non-trauma-associated ARDS has been addressed by matching. However, manual matching for a minority of variables has a risk of introducing bias itself. In addition, patients were matched based on parameters at ICU admission. Matching at the day of ARDS onset may have led to a different outcome, in case of a relevant time lag between ICU admission and ARDS onset. This was not the case, as most patients already presented with ARDS at ICU admission. Trauma-associated ARDS was a rare complication, which was observed in only 62 out of 1038 patients with ARDS in the 11-year period between 2007 and 2018 in this ARDS referral center. Due to the heterogeneity of patients and limited knowledge of relevant pre-clinical factors, such as pre-clinical rescue times, fluid resuscitation, or lowest body temperature, comparability between the present and the previously reported trauma-associated ARDS cohorts is difficult. Gender/sex-specific outcome analysis has not been performed due to the low number of female patients with trauma-associated ARDS. Thus, the conclusions are drawn from an imbalanced gender/sex data set, which may potentially contribute to gender bias. Due to the rarity of trauma-associated ARDS, properly designed prospective studies are nearly impossible to perform in this cohort. As a result, risk factors that can contribute to mortality in patients with trauma-associated ARDS and criteria for which trauma patients might benefit from treatment with ECLS must be clarified through high quality, retrospective multicenter studies. Consensus criteria for severity of trauma, ARDS, and multiorgan failure need to be consistently collected and reported to improve comparability of studies reporting data on patients with trauma-associated ARDS.

## 6. Conclusions

Mortality rate was not increased in patients with trauma-associated ARDS compared to patients with non-trauma-associated ARDS. This finding was observed in patients with trauma-associated ARDS and ECLS treatment, also. Although not significant, there were indications for time-sensitive differences in survival, particularly in the non-matched groups. Survival rate stabilized after the critical initial phase and throughout the 60-day period in patients with trauma-associated ARDS compared to patients with non-trauma-associated ARDS. Since this divergence was less pronounced in the matched cohort, this may be most likely related to the younger age, fewer comorbidities, and lower ARDS severity in patients with trauma-associated ARDS. Patients with trauma-associated ARDS presented with relevant differences in baseline characteristics and remain a very different cohort compared to patients with non-trauma-associated ARDS. Therefore, perfect matching was limited and the outcome comparison is not without caveats. Our results are explorative and do not allow for a confirmatory generalization.

## Figures and Tables

**Table 1:** Baseline Characteristics.

	A. Trauma- Associated ARDS *n* = 62	B1. Non-Trauma- Associated ARDS (all) *n* = 976	A vs. B1 *p*-Value	B2. Non-Trauma- Associated ARDS (matched) *n* = 62	A vs. B2 *p*-Value
Age (years)	45.0 (27.0/60.0)	53.0 (41.0/64.0)	0.003	44.5 (27.0/58.0)	0.383
Sex (f/m) (*n*, %)	11/51 (17.7/82.3)	351/625 (36.0/64.0)	0.004	11/51 (17.7/82.3)	1.00
SOFA at ICU admission	11.0 (10.0/14.0)	11.0 (8.0/14.0)	0.778	11.0 (9.0/13.0)	0.944
Oxygenation Index (OI)	11.9 (6.7/20.8)	15.5 (9.8/23.5)	0.009	12.3 (8.0/19.4)	0.546
Body mass index (kg/m^2^)	26.3 (24.7/30.2)	26.2 (22.9/31.3)	0.449	26.1 (23.6/28.6)	0.104
APACHE * II	24.0 (19.0/33.0)	26.0 (19.0/33.0)	0.404	25.5 (17.0/31.0)	0.760
TISS 28	51.0 (42.0/58.0)	48.0 (41.0/56.0)	0.077	47.0 (42.0/54.0)	0.110
SAPS II	47.5 (37.0/63.0)	55.0 (39.5/68.5)	0.051	53.0 (38.0/64.0)	0.053
Glasgow Coma Scale (GCS)	3.0 (3.0/3.0)	3.0 (3.0/3.0)	0.312	3.0 (3.0/3.0)	1.00
Septic shock *(n*,%)	37 (59.7)	430 (44.1)	0.022	31 (50.0)	0.458
Injury Severity Score (ISS)	29.0 (21.0/38.0)				
LOS ** in ICU of ARDS center (d)	21.4 (10.2/37.3)	17.0 (8.2/30.2)	0.118	18.7 (12.1/39.3)	0.853
Charlson Comorbidity Index	0.5 (0.0/2.0)	3.0 (1.0/5.0)	<0.001	2.0 (0.0/5.0)	<0.001
Cardiopulmonary resuscitation total (*n*,%)	20 (32.3)	270 (27.7)	0.434	20 (32.3)	1.00
Cardiopulmonary resuscitation extern (*n*,%)	11 (17.7)	137 (14.0)	0.418	9 (14.5)	0.804

Numerical variables are presented as median with limits of the interquartile range, categorical variables as number (percentage). * Acute physiology and chronic health evaluation, ** Length of stay. Missing values ≤10% were not reported, no missing values >10% were observed. Statistical Tests: Independent groups A vs. B1 Mann–Whitney Test for numerical variables, Pearson’s Chi^2^ for categorical variables. Matched cohort A vs. B2: Wilcoxon Test for numerical variables, McNemar’s Test for categorical variables.

**Table 2:** Primary Outcome Mortality.

	A. Trauma- Associated ARDS *n* = 62	B1. Non-Trauma- Associated ARDS (all) *n* = 976	A vs. B1 *p*-Value	B2. Non-Trauma- Associated ARDS (matched) *n* = 62	A vs. B2 *p*-Value
a					
28-day mortality (*n*,%)	16 (25.8)	299 (30.6)	0.423	18 (29.0)	0.845
60-day mortality (*n*,%)	17 (27.4)	373 (38.2)	0.089	19 (30.6)	0.845
In-hospital mortality (*n*,%)	18 (29.0)	395 (40.5)	0.074	21 (33.9)	0.701
b					
	**A. Trauma-** **Associated ARDS and ECLS *n* = 24**	**B1. Non-Trauma-** **Associated ARDS ** **and ECLS (all) *n* = 558**	**A vs. B1** ***p*** **-Value**	**B2. Non-Trauma-** **Associated ARDS and ECLS (matched)** ***n*** **= 29**	**A vs. B2** ***p*** **-Value**
28-day mortality in ECLS patients (*n*,%)	11 (45.8)	205 (36.7)	0.366	8 (27.6)	0.124
60-day mortality in ECLS patients (*n*,%)	11 (45.8)	267 (47.8)	0.846	8 (27.6)	0.124
In-hospital mortality in ECLS patients (*n*,%)	12 (50.0)	282 (50.5)	0.959	9 (31.0)	0.206

Numerical variables are presented as median with limits of the interquartile range, categorical variables as number (percentage). Statistical Tests: Independent groups A vs. B1 Mann–Whitney Test for numerical variables, Pearson Chi^2^ for categorical variables. Matched cohort A vs. B2: Wilcoxon Test for numerical variables, McNemar’s Test for categorical variables. Missing values ≤ 10% were not reported, no missing values >10% were observed.

**Table 3:** Severity of ARDS, gas exchange, mechanical ventilation settings, and supportive therapies.

	A. Trauma- Associated ARDS *n* = 62	B1. Non-Trauma- Associated ARDS (all) *n* = 976	A vs. B1 *p*-Value	B2. Non-Trauma-Associated ARDS (matched) *n* = 62	A vs. B2 *p*-Value
Mild: PaO_2_/FiO_2_ 201–300 mmHg, PEEP ≥5 cmH_2_O (*n*,%)	8 (12.9)	28 (2.9)	<0.001	2 (3.2)	0.149
Moderate: PaO_2_/FiO_2_ 101–200 mmHg, PEEP ≥5 cmH_2_O (*n*,%)	13 (21.0)	139 (14.2)		16 (25.8)	
Severe: PaO_2_/FiO_2_ ≤100 mmHg, PEEP ≥5 cmH_2_O (*n*,%) or patient with ECLS	41 (66.1)	809 (82.9)		44 (71.0)	
	Mechanical ventilation parameters in the first 6h after ICU admission				
P_max_ (cm H_2_O)	31.0 (25.5/36.0)	34.0 (30.5/37.9)	0.005	33.3 (30.6/37.0)	0.864
P_mean_ (cm H_2_O)	20.5 (16.5/25.5)	23.0 (20.0/26.0)	0.026	22.0 (19.0/28.0)	0.743
PEEP * (cm H_2_O)	14.7 (10.0/18.0)	16.3 (13.6/19.0)	0.019	16.4 (13.0/19.0)	0.218
Respiratory rate (n/min)	19.5 (16.5/22.0)	21.0 (18.0/24.0)	0.007	21.0 (18.0/23.0)	0.078
Respiratory volume (L/min)	9.2 (7.4/11.1)	8.3 (6.3/10.4)	0.019	8.8 (6.8/10.5)	0.217
FiO_2_ (%)	74.0 (60.5/90.0)	82.0 (70.0/94.0)	0.017	77.0 (68.0/91.0)	0.215
Pulmonary compliance (mL/cm H_2_O)	36.4 (30.0/52.3)	30.7 (20.8/43.1)	<0.001	34.3 (24.8/44.9)	0.172
	Pulmonary gas exchange in the first 6 h after ICU admission				
PaO_2_ (mmHg)	114.3 (91.1/147.2)	112.3 (83.3/152.2)	0.454	122.7 (90.9/176.6)	0.544
PaCO_2_ (mmHg)	44.0 (38.9/54.2)	51.6 (42.7/62.4)	<0.001	49.2 (39.7/61.2)	0.046
pH value	7.38 (7.28/7.44)	7.32 (7.24/7.39)	0.002	7.33 (7.26/7.41)	0.131
	Mechanical ventilation, ECLS and supportive therapies				
ECLS patients total (*n*,%)	24 (38.7)	558 (57.2)	0.005	29 (46.8)	0.424
ECMO (*n*,%)	15 (24.2)	402 (41.2)	0.108	20 (32.3)	0.271
ECLA (*n*,%)	8 (12.9)	97 (9.9)		6 (9.7)	
ECMO and ECLA	1 (1.6)	59 (6.0)		3 (4.8)	
NO inhalation (*n*,%)	36 (58.1)	683 (70.0)	0.049	38 (61.3)	0.839
Prone positioning (*n*,%)	34 (54.8)	661 (67.7)	0.036	45 (72.6)	0.052
Prone positions per patient	2.5 (2.0/7.0)	3.0 (2.0/5.0)	0.718	3.0 (2.0/6.0)	0.473
Side positioning 135° (*n*,%)	15 (24.2)	313 (32.1)	0.196	21 (33.9)	0.286
Side positions 135° per patient	1.0 (1.0/5.0)	2.0 (1.0/5.0)	0.288	2.0 (1.0/10.0)	0.043
Tracheotomy (*n*,%)	50 (80.6)	748 (76.6)	0.468	48 (77.4)	0.815
Spontaneous breathing achieved (*n*, %)	48 (77.4)	683 (70.0)	0.213	47 (75.8)	1.00

Numerical variables are presented as median with limits of the interquartile range, categorical variables as number (percentage). Statistical Tests: Independent groups A vs. B1 Mann–Whitney Test for numerical variables, Pearson’s Chi^2^ for categorical variables. Matched cohort A vs. B2: Wilcoxon Test for numerical variables, McNemar’s Test for categorical variables. * Positive End-Expiratory Pressure. Missing values ≤10% were not reported, no missing values >10% were observed.

**Table 4:** Transfusions.

	A. Trauma- Associated ARDS *n* = 62	B1. Non-Trauma- Associated ARDS (all) *n* = 976	A vs. B1 *p*-Value	B2. Non-Trauma-Associated ARDS (matched) *n* = 62	A vs. B2 *p*-Value
Number of patients who received transfusions					
Packed red blood cells transfused (*n*,%)	52 (83.9)	849 (87.0)	0.482	55 (88.7)	0.607
FFP transfused (*n*,%)	49 (79.0)	722 (74.0)	0.377	47 (75.8)	0.824
Platelet concentrates transfused (*n*,%)	33 (53.2)	426 (43.6)	0.141	30 (48.4)	0.720
Number of units of transfusions in patients who received transfusions					
Packed red blood cells until day 7 per patient (*n* per patient)	7.0 (4.0/13.0)	6.0 (3.0/10.0)	0.194	5.0 (2.0/9.0)	0.005
Packed red blood cells until day 14 per patient (*n* per patient)	8.0 (6.0/19.5)	8.0 (4.0/15.0)	0.326	6.0 (3.0/11.0)	0.014
Packed red blood cells until day 28 per patient (*n* per patient)	11.0 (6.0/27.0)	10.0 (5.0/20.0)	0.407	9.0 (4.0/18.0)	0.040
Packed red blood cells during ICU stay (*n* per patient)	12.5 (6.0/28.0)	11.0 (5.0/25.0)	0.357	10.0 (4.5/21.5)	0.099
FFP during ICU stay (*n* per patient)	28.0 (10.0/61.0)	21.0 (8.0/46.0)	0.170	19.0 (6.0/48.0)	0.015
Platelet concentrates (*n* per patient)	6.0 (3.0/13.0)	6.0 (2.0/13.0)	0.962	4.0 (2.0/14.0)	0.346

Numerical variables are presented as median with limits of the interquartile range, categorical variables as number (percentage). Statistical Tests: Independent groups A vs. B1 Mann–Whitney Test for numerical variables, Pearson’s Chi^2^ for categorical variables. Matched cohort A vs. B2: Wilcoxon Test for numerical variables, McNemar’s Test for categorical variables.

## Data Availability

For data protection reasons, public data disclosure is restricted. Editors, reviewers, and interested researchers may contact the corresponding author or send a request to dai-researchdata@charite.de to get data access.
